# Polyarthritis in Sjögren’s Syndrome: Difficulties in Distinguishing Extraglandular Manifestation and Associated Rheumatoid Arthritis

**DOI:** 10.3390/diagnostics14141494

**Published:** 2024-07-11

**Authors:** Zsófia Aradi, Gábor Nagy, Ildikó Fanny Horváth, Péter Antal-Szalmás, Antónia Szántó

**Affiliations:** 1 Division of Clinical Immunology, Institute of Internal Medicine, Faculty of Medicine, University of Debrecen, 4032 Debrecen, Hungary; aradi.zsofia@med.unideb.hu (Z.A.); fanny.horvath@med.unideb.hu (I.F.H.); 2 Department of Laboratory Medicine, Faculty of Medicine, University of Debrecen, 4032 Debrecen, Hungary; nagy.gabor@med.unideb.hu (G.N.); antalszp@med.unideb.hu (P.A.-S.)

**Keywords:** Sjögren’s syndrome, associated rheumatoid arthritis, polyarthritis, extraglandular manifestation

## Abstract

Aim of the study was to investigate the demographic data and disease course characteristics of patients with Sjögren’s syndrome (SS) and inflammatory joint pain of various origins and to search for factors that might help with the distinction of polyarthritis as an extraglandular manifestation and rheumatoid arthritis as an associated systemic autoimmune disorder. A total of 355 patients were retrospectively analyzed, 128 of whom served as controls (SS-C), while 159 had polyarthritis as an extraglandular symptom of Sjögren’s syndrome (SS-pa) and 68 were diagnosed as having associated rheumatoid arthritis (SS-RA). The patients without any inflammatory joint manifestations were significantly older than the SS-pa patients, while, for the SS-RA group, the difference was not significant. The onset of joint pain appeared significantly earlier in the SS-RA patients. Regarding either extraglandular manifestations or associated autoimmune disorders, there were significant differences between the controls and both SS-pa and SS-RA groups, while no significant difference was found between the SS-pa and SS-RA groups. Thus, laboratory and imaging methods should be used to differentiate between the two conditions, but laboratory biomarkers are even more important for early diagnosis. A ROC curve analysis showed an acceptable diagnostic accuracy in differentiating between SS-pa and SS-RA patients using a binary logistic regression model, where highly positive rheumatoid factor (RF) and anti-cyclic citrullinated peptide (CCP) values, kidney involvement, and anti-Ro/SS-A positivity were shown to significantly raise the odds of having RA, whereas anti-La/SS-B positivity seemed to have a protective role, since it significantly decreased the odds of having it. Further biomarkers are needed to better classify SS patient cohorts with inflammatory joint pain of different origins and, consequently, different management requirements.

## 1. Introduction

Sjögren’s syndrome (SS) is a systemic autoimmune disease affecting primarily the exocrine glands but involving several other organs, as well. The most prominent symptoms that are present in the vast majority of patients include dry eyes, dry mouth, fatigue, and joint pain. The clinical presentation and severity of symptoms can vary widely between individuals, ranging from few or mild symptoms to unbearable dryness, pain, or disabling fatigue [1]. In approximately 30–40% of Sjögren’s patients, the disease is accompanied by systemic manifestations and can cause the dysfunction of various organs, such as the lungs, liver, kidneys, gastrointestinal tract, joints, muscles, and peripheral or central nervous system. Patients also have a higher risk of developing B cell lymphoma [2]. The risk of B cell lymphoma is 15 to 20 times higher among adult patients with SS compared to the general population (lifetime risk: 5–10%) [3]. The disease affects mainly women, with a female/male ratio of 9:1, and the onset may occur at any age [4]. Antibodies characteristic for the disease are anti-Ro/Sjögren’s syndrome-A (anti-Ro/SS-A) and anti-La/Sjögren’s syndrome-B (anti-La/SS-B).

Articular involvement is the most common extraglandular manifestation. According to the study of Fauchais et al., approximately 30–60% of primary SS patients suffered from articular manifestations, which were associated with multisystem involvement [5]. Joint manifestations can be grouped as osteoarthritis, being predominantly age-related and, therefore, not requiring immunomodulant medications, despite the pain, as a common feature; non-erosive polyarthritis as an extraglandular manifestation of SS; and rheumatoid arthritis (RA) as another systemic autoimmune disease associated with SS. According to a recent paper by Gao and colleagues, the most common cause of joint pain is either osteoarthritis, especially knee osteoarthritis, or joint involvement due to the disease itself: SS-polyarthritis. They also proved that primary SS patients with advanced age and more pronounced metabolic characteristics, such as elevated blood lipid and uric acid levels, are at risk for osteoarthritis. Moreover, SS-polyarthritis patients had higher disease activity and more organs involved [6]. Another study showed that almost half of the anti-citrullinated protein antibody (ACPA)-positive patients with SS developed RA during their disease course. The absence of joint destruction and bone erosions distinguishes SS-polyarthritis from RA, where joint damage more frequently occurs and is a disease hallmark [7].

It is of great importance to differentiate between the two types of inflammatory joint manifestations in a single patient, since the association with RA requires an earlier and more aggressive disease-modifying antirheumatic drug (DMARD) or even further escalated, targeted treatment to prevent the development of irreversible erosions [8].

In our study, we aimed to compare the characteristic demographic, clinical, and laboratory parameters of SS patients with different kinds of inflammatory joint manifestations in order to search for potential factors that help in distinguishing them.

## 2. Materials and Methods

We retrospectively reviewed the data of a total of 355 patients with Sjögren’s syndrome being regularly followed up at the Division of Clinical Immunology, Faculty of Medicine, University of Debrecen, Hungary. Patients presenting at least once during the year 2019 were included. In total, 227 (63.9%) patients were identified to have some kind of inflammatory joint involvement. They were further divided into two groups according to having polyarthritis complicating Sjögren’s syndrome as an extraglandular manifestation (SS-pa, *n* = 159; 47.4%) or having rheumatoid arthritis associated with Sjögren’s syndrome (SS-RA, *n* = 68; 19.15%). The patients were classified according to the American College of Rheumatology (ACR)—European League Against Rheumatism (EULAR) criteria for Sjögren’s syndrome [9]—and for rheumatoid arthritis [8], keeping in mind that the latter criteria set was developed to recognize early rheumatoid arthritis. Therefore, and because of the overlapping features of the two systemic autoimmune diseases potentially resulting in differential diagnostic issues, the erosive nature of joint involvement was proved with imaging procedures. A comparative X-ray examination of the hands was performed in all cases, while, in some patients, the small joints of the feet were imaged, too. In cases where no erosive lesions were found, but they were suspected by clinical symptoms, MR was performed to detect potential abnormalities for which X-ray is not feasible. Patients without any inflammatory joint complaints served as the control group (SS-C, *n* = 128; 36.0%). Since we aimed to characterize inflammatory joint manifestations, the patients with osteoarthritis were merged into the control group, where patients might have had any other glandular or extraglandular features except for inflammatory joint manifestations. The patient groups were compared according to their demographic data, laboratory parameters, associated diseases, and treatment modalities. Organic manifestations (such as lung, kidney, cutaneous involvement, and lymphadenopathy) were defined according to the EULAR Sjögren’s Syndrome Disease Activity Index (ESSDAI) domains [10]. Laboratory parameters were determined as part of the general routine investigation and follow-up of the involved patients at the Department of Laboratory Medicine. C-reactive protein, rheumatoid factor IgM, and total immunoglobulin G concentrations were measured using turbidimetry (Cobas c503 clinical chemistry analyzer, Roche Diagnostics, Basel, Switzerland). Antinuclear antibodies were tested using HEp-2 indirect immunofluorescence assay (FC 1522–1010 ANA HEp 20–10 EUROPattern, Euroimmun, Lübeck, Germany), while enzyme-linked immunosorbent assay (ELISA) kits were used for anti-cyclic citrullinated peptide (anti-CCP) (RA96Plus Immunoscan RA anti-CCP IgG, Svar Life Science, Malmö, Sweden), anti-Ro/SS-A, and anti-La/SS-B (EA 1595–9601G SS-A(Ro) IgG and EA 1597–9601G SSB(La) IgG, Euroimmun, Lübeck, Germany) measurement.

### Statistical Analysis

SPSS software (version 24.0) was used for statistical analysis. The Kolmogorov–Smirnov test was used for the evaluation of normality. For continuous parameters not showing a normal distribution, Kruskal–Wallis and Mann–Whitney tests were carried out, whereas, for those with normal distribution, analysis of variance (ANOVA) and two-sample *T* tests were used. For discrete parameters, the Fisher’s exact test was used when the expected count was <5, while the chi-square test was performed when the expected data were >5. *p* values < 0.05 were considered statistically significant.

For a more detailed analysis, a binary logistic regression model was created to show which parameters are independent predictors. Where necessary, continuous parameters were converted into dichotomous groups using cut-off values respecting the normal laboratory values. Receiver operating characteristic (ROC) analysis was performed to detect differential diagnostic accuracy in distinguishing between the three patient groups. The area under the curve (AUC) and its confidence interval are reported, where a higher AUC means better discrimination and a diagonal line (AUC = 0.5) indicates no differentiation.

## 3. Results

### 3.1. Age and Gender

The male/female ratio was 13/116 in the SS-C group, 8/151 in SS-pa, and 3/65 in the SS-RA subset. The median age of the patients was highest in the SS-C group (68 years), and the difference was significant compared to the SS-pa group, where patients were the youngest (63 years). In the SS-RA group, the median age was 65.5 years, and the difference was not significant compared to the other groups (Table 1).

### 3.2. Laboratory Parameters

**Figure 1:**
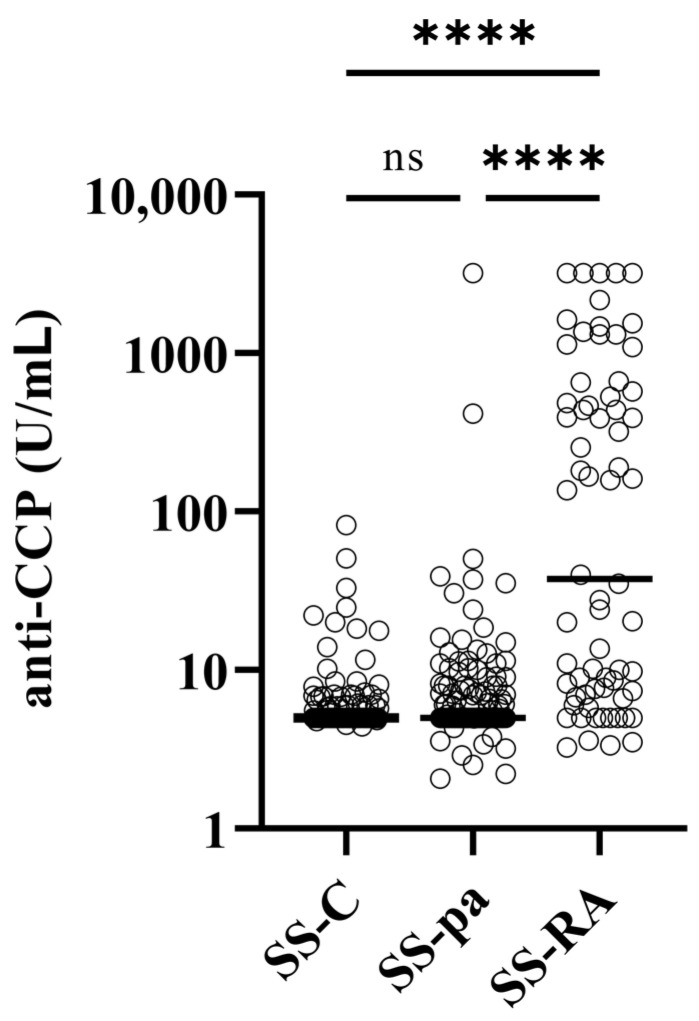
Anti-CCP levels of the patient groups. Abbreviations: anti-CCP: anti-cyclic citrullinated peptide; SS-C: Sjögren’s syndrome controls; SS-pa: Sjögren’s syndrome with polyarthritis; SS-RA: Sjögren’s syndrome associated with rheumatoid arthritis, ns: not significant, ****: *p* < 0.0001, reference range: <25 U/mL.

**Figure 2:**
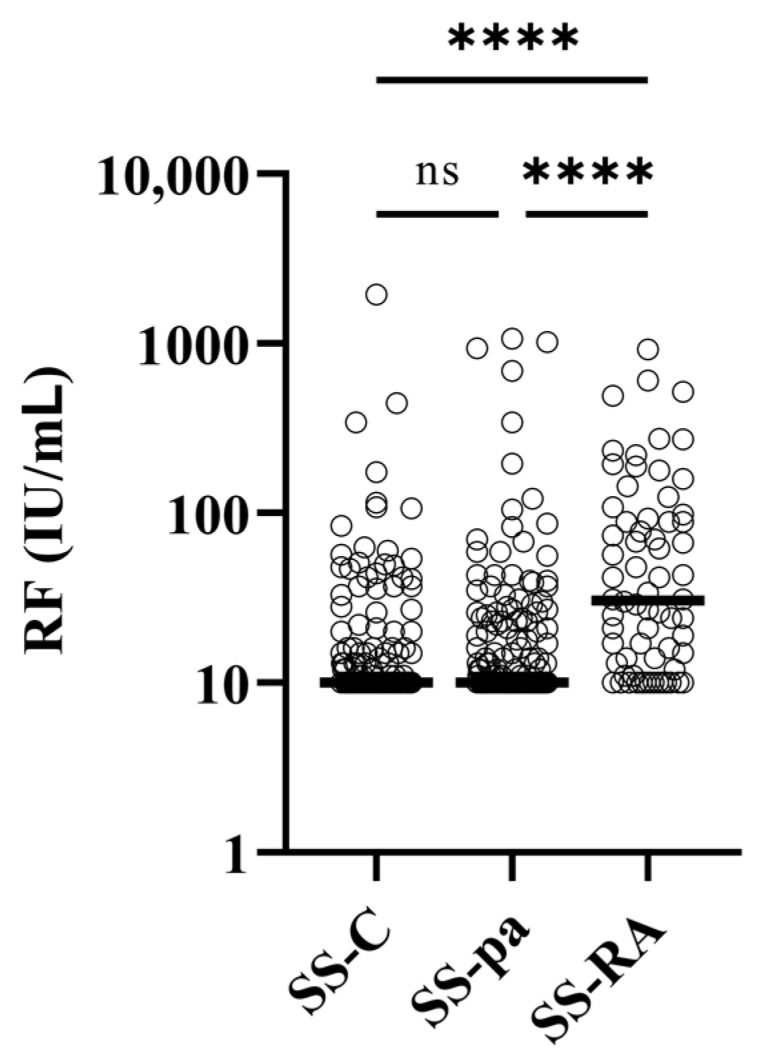
Rheumatoid factor levels in the patient groups. Abbreviations: RF: rheumatoid factor; SS-C: Sjögren’s syndrome controls; SS-pa: Sjögren’s syndrome with polyarthritis; SS-RA: Sjögren’s syndrome associated with rheumatoid arthritis, ns: not significant, ****: *p* < 0.0001, reference range: <14 IU/mL.

No significant differences were found between the three patient groups regarding C-reactive protein, leukocyte count, or erythrocyte sedimentation rate. Considering the immunological parameters, there was no significant difference regarding the IgG levels, occurrence of ANA, anti-Ro/SS-A, or anti-La/SS-B levels or frequency (Table 1). Both anti-CCP (Figure 1) and rheumatoid factor (Figure 2) levels were significantly higher in the SS-RA group than in SS-pa or SS-C patients, while the differences between the latter groups were not significant.

### 3.3. Extraglandular Manifestations and Associated Diseases

Reviewing the time passed from the diagnosis of SS until the onset of joint complaints, we found that joint complaints evolved significantly earlier in the SS-RA group (−1.18 ± 6.11 years, where a negative value means an onset before the diagnosis of SS) than in the SS-pa group (1.3 ± 5.8 years) (*p* = 0.004).

The occurrence of lymphadenopathy did not show significant differences between the three patient groups. Raynaud’s phenomenon was significantly more frequent in the patients with polyarthritis compared to the control group. Lung involvement was most frequent in the SS-RA patients, and the difference was significant compared to the SS-C group. Moreover, the SS-pa patients had lung manifestations significantly more often than the SS-C group, as well, however, there was no significant difference between the SS-RA and SS-pa patients (Table 1). Kidney involvement occurred in a significantly smaller proportion of the SS-C group than in the SS-RA group. The occurrence of cutaneous manifestations defined as palpable purpura, recurrent urticaria, and photosensitivity, as well as the frequency of Hashimoto’s thyroiditis, did not differ among the three patient groups (Table 1). Regarding the association of further systemic and organ-specific autoimmune diseases (systemic lupus erythematosus, antiphospholipid syndrome, celiac disease, and primary biliary cholangitis) and the occurrence of non-immune diseases (hypertension, type 2 diabetes mellitus, and chronic obstructive pulmonary disorder), the patient groups did not differ (Table 1).

### 3.4. Treatment

The drugs used for the management of patients are summarized in Table 2. Among the therapies used, sulfasalazine and antimalarial use was significantly more frequent in the SS-pa patients when compared to the SS-C group, but no significant difference was detected between the SS-pa and SS-RA groups or the SS-RA and SS-C groups. The SS-RA patients required glucocorticoids significantly more often during their disease course than any other patient group. Methotrexate use was gradually significantly less common in the control group than in the two groups with inflammatory joint manifestations, while this therapy was more frequent in the SS-RA patients than in the SS-pa group. Leflunomide was used exclusively in the patients with rheumatoid arthritis.

### 3.5. Binary Multiparametric Logistic Regression Model and ROC Curve Analysis

After developing subsets based on having low-positive (≤3 × upper limit of normal—ULN) or high-positive (≥3 × ULN) anti-CCP and rheumatoid factor levels, a binary multiparametric logistic regression model was created. This model provided further diagnostic value in differentiating the three patient groups. It became clear that only high-positive anti-CCP and RF levels can increase the accuracy of the model, whereas—unlike anti-Ro/SS-A—anti-La/SS-B positivity decreases the chance of having SS-RA. A high IgG level is another laboratory parameter associated with lower odds for RA when compared to control patients. Among the organic manifestations, kidney involvement was able to improve the diagnostic accuracy of differentiating SS-RA from SS-pa. Raynaud’s syndrome and lung involvement were proved to be independent factors related to joint disease by this multiparametric approach (Table 3).

**Figure 3:**
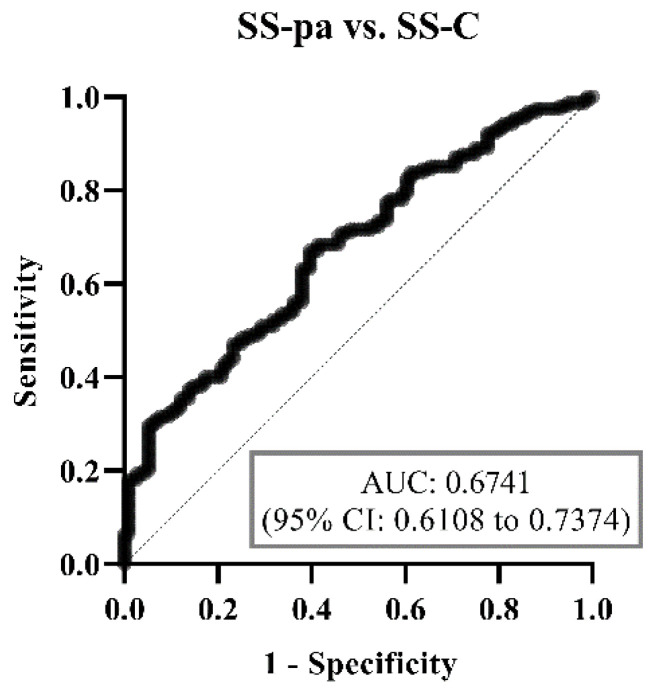
ROC curve analysis of binary logistic regression model regarding SS-pa vs. SS-C groups. Abbreviations: SS-C: Sjögren’s syndrome controls; SS-pa: Sjögren’s syndrome with polyarthritis; SS-RA: Sjögren’s syndrome associated with rheumatoid arthritis, AUC: area under curve, CI: confidence interval.

**Figure 4:**
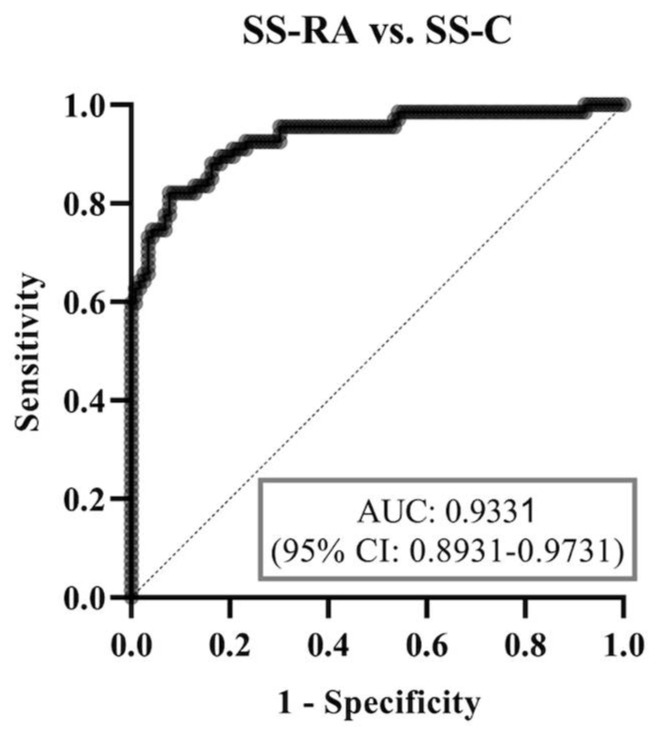
ROC curve analysis of binary logistic regression model regarding SS-RA vs. SS-C groups. Abbreviations: SS-C: Sjögren’s syndrome controls; SS-pa: Sjögren’s syndrome with polyarthritis; SS-RA: Sjögren’s syndrome associated with rheumatoid arthritis, AUC: area under curve, CI: confidence interval.

**Figure 5:**
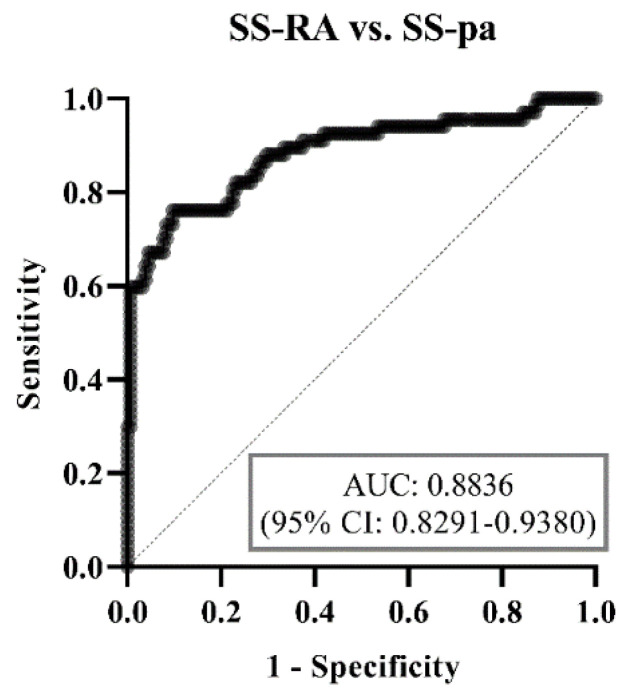
ROC curve analysis of binary logistic regression model regarding SS-pa vs. SS-RA groups. Abbreviations: SS-C: Sjögren’s syndrome controls; SS-pa: Sjögren’s syndrome with polyarthritis; SS-RA: Sjögren’s syndrome associated with rheumatoid arthritis, AUC: area under curve, CI: confidence interval.

Using ROC analysis, the diagnostic accuracy of the binary multiparametric logistic regression model was weakest in the SS-C vs. SS-pa patients (area under curve (AUC) = 0.6741, Figure 3), best at distinguishing between the SS-C and SS-RA groups (AUC = 0.9331, Figure 4). Regarding the most crucial issue from a physician’s perspective, differentiating SS-pa patients from SS-RA patients, the diagnostic accuracy was good (AUC = 0.8836, Figure 5), and, what is even more important, it was better than that of the anti-CCP test alone (AUC = 0.8083, ROC curve not shown).

## 4. Discussion

Arthralgia and non-erosive polyarthritis affecting small joints are frequent extraglandular symptoms of Sjögren’s syndrome. It has long been a debate as to how to distinguish these non-aggressive, non-erosive manifestations from early rheumatoid arthritis [11,12,13]. In such cases, the rheumatoid factor does not help us to differentiate between the two diseases, as it is not specific enough for rheumatoid arthritis and is often found in patients with Sjögren’s syndrome. A parallel examination of anti-CCP and rheumatoid factor is recommended, since higher anti-CCP levels have been detected more often in Sjögren’s syndrome associated with rheumatoid arthritis than in primary Sjögren’s syndrome [14].

We retrospectively analyzed the data of a total of 355 patients, out of which 227 had inflammatory joint involvement. We found a significant difference regarding the time until the onset of the joint complaints between the SS-pa and SS-RA patients (15.6 vs. −14.16 months, respectively), meaning that, in the latter group, joint pain might precede the diagnosis of SS. In a follow-up examination by Ryu et al., the mean duration of progression of anti-CCP positive primary SS patients to RA was 60 months [15]. Our results suggest that high anti-CCP levels in SS patients with inflammatory joint pain indicate progression towards rheumatoid arthritis. This finding corresponds to the observation of the above-mentioned study, since they found that the anti-CCP antibody titer was independently associated with the progression to RA [15].

Based on our results, the occurrence of certain organ manifestations differs in the three groups. Pulmonary involvement occurred significantly more often in the groups with inflammatory joint manifestations than in the SS-C patients. In another study, where ACPA-negative and ACPA-positive SS patients were compared, pulmonary involvement presented significantly more frequently in the ACPA-positive than in ACPA-negative SS patients, as well (4/16 vs. 22/278, respectively) [7]. Similar results were found in another cohort, where SS-RA patients were compared to SS patients, regardless of having polyarthritis or not [16]. Lung involvement was more frequent, and the RF and anti-CCP levels were higher in the cohort of SS-RA patients compared to SS, however, this paper focused on the distinction of RA, SS, and SS-RA. They found that the SS-RA patients had more severe arthritis than the RA patients; moreover, rash, fever, and hematological abnormalities were also more frequent [17].

Regarding organ-specific autoimmune diseases, Hashimoto’s thyroiditis was present at a considerable rate in patients of each group, without any significant difference. Our findings correspond to others, concluding that Hashimoto’s thyroiditis is significantly more common in both primary SS and RA patients than in the general population [18,19].

Renal involvement occurred significantly more often in the SS-RA group than in the SS-C patients. Based on the literature data, kidney involvement in Sjögren’s syndrome is rather infrequent, affecting less than 10% of patients, and presents mainly as tubulointerstitial nephritis, or, even less frequently, as membranoproliferative glomerulonephritis [20,21], whereas renal manifestations in RA have gradually evolved parallelly with the improvement of the management of the disease [22].

There was no significant difference regarding the occurrence of cutaneous manifestations between the individual groups. Our data correspond with the findings of Soy et al., reporting that almost half of Sjögren’s syndrome patients manifest various skin symptoms during their disease course [23].

A significantly larger proportion of SS-RA patients require glucocorticoid treatment than either of the other two groups. This can be explained by the fact that glandular symptoms (SS-C group) usually do not need systemic treatment, while pain and inflammation in non-erosive polyarthritis are usually less severe than those observed in rheumatoid arthritis. Sulfasalazine and antimalarials are significantly more often used in SS-pa patients than in the SS-C group, whereas no significant difference was found between either the SS-RA and SS-pa patients or between the SS-RA and SS-C groups, meaning that these immunomodulant agents were predominantly used to manage mild forms of polyarthritis complicating SS [24]. Unsurprisingly, methotrexate use was gradually and significantly more frequent in the SS-C, SS-pa, and SS-RA patients, respectively.

Our results draw attention to the importance of the regular and systematic follow-up of patients with Sjögren’s syndrome, since polyarthritis, as a symptom, may suggest the progression to rheumatoid arthritis, especially when inflammatory joint complaints precede the diagnosis of SS or even sicca symptoms. Yang et al. found that the presence of arthritis, RF, and anti-CCP were independent risk factors for SS overlapping with RA [25]. The distinction between patients with SS-pa and SS-RA is of highest importance, since it outlines the therapeutic consequences. Since there is no significant difference between these two patient groups regarding the occurrence of either extraglandular manifestations or associated organ-specific autoimmune diseases, it is difficult for clinicians to decide the exact origin of arthritis [16] in these patients.

From a different point of view, recognizing the association of SS in patients with RA is also important. The prevalence of SS in RA patients was found to be 8.7–10% by different groups [26,27].

According to a recent paper, even the responsiveness to targeted treatments is different in RA patients when associated with Sjögren’s syndrome, as follows: anti-tumor-necrosis factor (TNF)-alpha agents are less effective, whereas rituximab is more effective than in RA alone [28]. Furthermore, joint damage is worse in SS-RA patients than in RA patients without the association of SS [29,30]. Several authors highlight the importance of recognizing erosive arthritis during the disease course of SS [17,29,31], being most beneficial before the emergence of irreversible radiological manifestations [31]. Our ROC curve analysis could achieve an acceptable differential diagnostic accuracy, yet it cannot be easily used in everyday practice for individual cases. Thus, laboratory examinations must take priority. As supported by our results, rheumatoid factor assessment is not enough to distinguish between early rheumatoid arthritis and Sjögren’s syndrome complicated by polyarthritis. If clinical suspicion is high, the measurement of anti-citrullinated protein antibodies is highly recommended. In both cases, high-positive (≥3 × ULN) values improve diagnostic accuracy. Nevertheless, in patients with Sjögren’s syndrome complicated by polyarthritis, awareness justifies regular anti-CCP screening, until a more suitable biomarker appears on the horizon.

This study has the following limitations: the data were collected retrospectively, and no disease activity scores were calculated, due to the overlapping features of SS and RA.

## 5. Conclusions

During the follow-up of patients with SS, the recognition of associated rheumatoid arthritis at the right time helps to provide an appropriate treatment and thereby slow down or prevent the progression of bone erosions. However, in everyday practice, it is not always simple to recognize the exact background of recent-onset polyarthritis in a patient with long-standing SS. In our binary multiparametric logistic regression model, using high-positive (≥3 × ULN) RF and anti-CCP levels, an acceptable level of differential diagnostic accuracy was achieved, where kidney involvement and anti-Ro/SS-A positivity further increased the chance of having SS-RA, and, on the contrary, anti-La/SS-B positivity decreased it. However, this model still does not perfectly predict the co-existence of RA in the background of inflammatory arthritis in an SS patient. Therefore, further biomarkers should be found for an easier distinction between the different origins of inflammatory joint manifestations during the disease course of individuals with SS.

## Figures and Tables

**Table 1:** Characteristics of patient groups (SS-C: Sjögren’s syndrome control patients, SS-pa: Sjögren’s syndrome patients with polyarthritis, SS-RA: patients with the association of Sjögren’s syndrome and rheumatoid arthritis, CCP: cyclic citrullinated peptide, RF: rheumatoid factor, ANA: antinuclear antibody, CRP: C-reactive protein, ESR: erythrocyte sedimentation rate, WBC: white blood cell, IgG: immunoglobulin G). The laboratory test results are presented as the median value determined in each subgroup. Reference ranges: anti-CCP < 25 U/mL, RF: <14 IU/mL, anti-Ro/SS-A: <10 U/mL, anti-La/SS-B < 10 U/mL, CRP: <5 mg/L, ESR < 20 mm/h, WBC: 4.5–10.8 G/L, IgG: 7–16 g/L.

Parameter	SS-C *n* = 129	SS-pa *n* = 159	SS-RA *n* = 68	SS-pa vs. SS-C	SS-RA vs. SS-C	SS-RA vs. SS-pa
Male gender	13 (10.2%)	8 (5%)	3 (4.4%)	0.1133	0.2719	1.0000
Age (median year)	68.0	63.0	65.5	**0.0140**	1.0000	0.2273
Anti-CCP (U/mL)	5	5	37.5	1.0000	**<0.0001**	**<0.0001**
Anti-CCP positivity	3 (2.3%)	7 (4.4%)	36 (52.9%)	0.5223	**<0.0001**	**<0.0001**
RF (IU/mL)	10	10	30.5	1.0000	**<0.0001**	**<0.0001**
RF positivity	45 (35.4%)	54 (34.2%)	48 (70.6%)	0.8248	**<0.0001**	**<0.0001**
ANA positivity	70 (54.7%)	94 (59.1%)	40 (58.8%)	0.4507	0.5786	0.9669
Anti-Ro/SS-A (U/mL)	10	10	10	1.0000	1.0000	1.0000
Anti-Ro/SS-A positivity	49 (38.3%)	65 (40.9%)	26 (38.2%)	0.6546	0.9950	0.7095
Anti-La/SS-B (U/mL)	10	10	10	1.0000	0.3889	0.4855
Anti-La/SS-B positivity	34 (26.6%)	44 (27.7%)	12 (17.6%)	0.8335	0.1610	0.1085
CRP (mg/L)	2.6	2.2	2.6	1.0000	1.0000	1.0000
CRP positivity	30 (23.4%)	43 (27%)	23 (33.8%)	0.4990	0.1308	0.3395
ESR (mm/h)	20	18	22	1.0000	0.8268	0.3462
ESR positivity	70 (54.7%)	79 (49.7%)	41 (60.3%)	0.4083	0.5450	0.1499
WBC (G/L)	6.15	6.25	6.15	1.0000	1.0000	1.0000
WBC abnormal%	25 (19.5%)	34 (21.4%)	16 (23.5%)	0.6656	0.5677	0.9297
IgG (g/L)	10.7	10.7	10.3	1.0000	0.2748	0.7956
IgG abnormal%	32 (25%)	31 (19.5%)	12 (17.6%)	0.4397	0.1305	0.3840
Raynaud’s phenomenon	27 (21.1%)	52 (33.3%)	19 (27.9%)	**0.0245**	0.2929	0.4417
Lung involvement	10 (7.8%)	25 (16.4%)	15 (22.1%)	**0.0323**	**0.0065**	0.3473
Hashimoto’s thyroiditis	28 (21.9%)	22 (13.8%)	11 (16.2%)	0.0857	0.4524	0.6828
Kidney involvement	7 (5.5%)	16 (10.1%)	10 (14.9%)	0.1914	**0.0335**	0.3611
Lymphadenopathy	4 (3.1%)	9 (5.7%)	4 (5.9%)	0.3978	0.4531	1.0000
Skin involvement	33 (25.8%)	52 (32.7%)	25 (36.8%)	0.2419	0.1388	0.6464
Associated autoimmune diseases	39 (30.5%)	52 (33.3%)	19 (27.9%)	0.6137	0.7451	0.4417
Associated non-autoimmune disease	45 (35.2%)	60 (38.4%)	21 (30.9%)	0.6233	0.6344	0.2959

**Table 2:** Drugs used for the management of patients (SS-C: Sjögren’s syndrome control patients, SS-pa: Sjögren’s syndrome patients with polyarthritis, SS-RA: patients with association of Sjögren’s syndrome and rheumatoid arthritis).

Parameter	SS-C *n* = 129	SS-pa *n* = 159	SS-RA *n* = 68	SS-pa vs. SS-C	SS-RA vs. SS-C	SS-RA vs. SS-pa
Glucocorticoid	35 (27.3%)	51 (32.1%)	50 (73.5%)	0.4373	**<0.0001**	**<0.0001**
Methotrexate	4 (3.1%)	20 (12.6%)	25 (36.8%)	**0.0045**	**<0.0001**	**<0.0001**
Leflunomid	0 (0%)	0 (0%)	25 (36.8%)	-	-	-
Sulfasalazine	3 (2.3%)	14 (8.8%)	3 (4.4%)	**0.0236**	0.4198	0.4085
Azathioprine	8 (6.3%)	17 (10.7%)	2 (2.9%)	0.2113	0.4986	0.0666
Antimalarials	24 (18.8%)	49 (30.8%)	17 (25%)	**0.0209**	0.3571	0.4272

**Table 3:** Binary multiparametric regression model with odds ratios. (SS-C: Sjögren’s syndrome control patients, SS-pa: Sjögren’s syndrome patients with polyarthritis, SS-RA: patients with association of Sjögren’s syndrome and rheumatoid arthritis, CCP: cyclic citrullinated peptide, RF: rheumatoid factor, CRP: C-reactive protein, ESR: erythrocyte sedimentation rate, WBC: white blood cell, IgG: immunoglobulin G).

	SS-pa vs. SS-C		SS-RA vs. SS-C		SS-RA vs. SS-pa	
Parameter	Odds Ratio	*p* Value	Odds Ratio	*p* Value	Odds Ratio	*p* Value
Age	1 (0.99–1.01)	0.5414	**0.96 (0.94–0.98)**	**<0.0001**	**0.97 (0.95–0.98)**	**<0.0001**
Anti-CCP low positive	2.04 (0.36–15.93)	0.4387	9.49 (0.64–245.1)	0.115	4.49 (0.63–30.02)	0.1181
Anti-CCP high positive	1.73 (0.11–48.51)	0.7043	**419.3 (42.67–14,068)**	**<0.0001**	**164.6 (31.68–1461)**	**<0.0001**
RF low positive	0.93 (0.43–2.03)	0.8525	2.49 (0.59–10.69)	0.211	2.06 (0.65–6.25)	0.2057
RF high positive	1.27 (0.48–3.39)	0.6283	**18.73 (3.91–111.5)**	**0.0005**	**13.5 (3.87–51.97)**	**<0.0001**
Anti-SS-A/Ro60 positive	1.74 (0.73–4.3)	0.2176	**5.92 (1.27–30.72)**	**0.0267**	**6.04 (1.74–22.73)**	**0.0057**
Anti-SS-B/La positive	0.73 (0.28–1.87)	0.509	**0.06 (0.01–0.36)**	**0.0035**	**0.05 (0.01–0.2)**	**<0.0001**
Raynaud’s syndrome	**2.01 (1.12–3.68)**	**0.0206**	**3.42 (1.13–11.06)**	**0.0328**	1.63 (0.65–4.19)	0.2997
Lung involvement	**3.02 (1.2–8.44)**	**0.0246**	**11.55 (2.14–71.76)**	**0.0057**	1.36 (0.4–4.34)	0.611
Hashimoto’s thyreoditis	0.61 (0.3–1.21)	0.1574	0.77 (0.2–2.68)	0.686	1.9 (0.59–5.75)	0.2644
Kidney involvement	1.65 (0.59–4.93)	0.3477	3.42 (0.63–18.89)	0.1491	**4.67 (1.07–20.06)**	**0.037**
Lymphadenopathy	1.86 (0.43–9.95)	0.4251	0.18 (0–14.55)	0.4396	0.14 (0.01–1.06)	0.0697
Skin involvement	1.28 (0.7–2.35)	0.4291	1.46 (0.44–4.59)	0.5198	0.72 (0.28–1.76)	0.4753
Associated immune diseases	1.05 (0.58–1.9)	0.8693	0.73 (0.19–2.63)	0.6361	0.68 (0.25–1.78)	0.4446
Associated non-immune diseases	1.03 (0.59–1.82)	0.9096	1.56 (0.47–5.23)	0.4647	1.14 (0.45–2.86)	0.7746
Male gender	0.53 (0.18–1.49)	0.2351	0.07 (0–1.01)	0.1148	0.28 (0.02–2.33)	0.2953
CRP positive	0.84 (0.43–1.65)	0.6141	1.03 (0.31–3.32)	0.9647	1.12 (0.4–3.05)	0.8207
ESR positive	0.79 (0.43–1.45)	0.4556	1.21 (0.31–4.86)	0.7792	1.03 (0.4–2.62)	0.9594
WBC low	0.79 (0.36–1.72)	0.5451	0.3 (0.05–1.34)	0.1355	1.17 (0.32–4.07)	0.8108
WBC high	2.41 (0.68–10.07)	0.1903	1.21 (0.03–20.06)	0.9015	0.31 (0.02–2.78)	0.3548
IgG low	0.42 (0.16–1.08)	0.0734	0.67 (0.08–4.09)	0.6825	0.43 (0.06–2.23)	0.3391
IgG high	0.83 (0.3–2.26)	0.7093	**0.03 (0–0.25)**	**0.0028**	0.23 (0.03–1.27)	0.1091

## Data Availability

Dataset available on request from the authors.
